# Nerve regeneration by human corneal stromal keratocytes and stromal fibroblasts

**DOI:** 10.1038/srep45396

**Published:** 2017-03-28

**Authors:** Gary Hin-Fai Yam, Geraint P. Williams, Melina Setiawan, Nur Zahirah Binte M. Yusoff, Xiao-wen Lee, Hla Myint Htoon, Lei Zhou, Matthias Fuest, Jodhbir S. Mehta

**Affiliations:** 1 Tissue Engineering and Stem Cell Group, Singapore Eye Research Institute, Singapore; 2 Ophthalmology and Visual Science Academic Clinical Research Program, Duke-National University of Singapore Graduate Medical School, Singapore; 3 Cornea and External Eye Disease Service Team, Singapore National Eye Centre, Singapore; 4 Singapore Eye Research Institute, Singapore; 5 Department of Ophthalmology, Yong Loo Lin School of Medicine, National University of Singapore, Singapore; 6 Department of Ophthalmology, RWTH Aachen University, Aachen, Germany; 7 School of Materials Science and Engineering, Nanyang Technological University, Singapore

## Abstract

Laser refractive surgeries reshape corneal stroma to correct refractive errors, but unavoidably affect corneal nerves. Slow nerve regeneration and atypical neurite morphology cause desensitization and neuro-epitheliopathy. Following injury, surviving corneal stromal keratocytes (CSKs) are activated to stromal fibroblasts (SFs). How these two different cell types influence nerve regeneration is elusive. Our study evaluated the neuro-regulatory effects of human SFs versus CSKs derived from the same corneal stroma using an *in vitro* chick dorsal root ganglion model. The neurite growth was assessed by a validated concentric circle intersection count method. Serum-free conditioned media (CM) from SFs promoted neurite growth dose-dependently, compared to that from CSKs. We detected neurotrophic and pro-inflammatory factors (interleukin-8, interleukin-15, monocyte chemoattractant protein-1, eotaxin, RANTES) in SFCM by Bio-Plex Human Cytokine assay. More than 130 proteins in SFCM and 49 in CSKCM were identified by nanoLC-MS/MS. Proteins uniquely present in SFCM had reported neuro-regulatory activities and were predicted to regulate neurogenesis, focal adhesion and wound healing. Conclusively, this was the first study showing a physiological relationship between nerve growth and the metabolically active SFs versus quiescent CSKs from the same cornea source. The dose-dependent effect on neurite growth indicated that nerve regeneration could be influenced by SF density.

The human cornea is one of the most richly innervated tissues in the body and corneal sensation is essential for corneal function^1^. The majority of corneal nerves are sensory and derived from the ophthalmic branch of trigeminal nerve^2^. The nerve bundles enter the cornea at the periphery in a radial fashion and run parallel to the corneal surface with repetitive branching to form a stromal nerve network^3^. Nerves in the most anterior stroma bend at right angles to penetrate Bowman’s layer to enter the corneal epithelium and subdivide into interconnected branches to form the sub-basal nerve plexus. Mixtures of straight and beaded nerve fibers then protrude between adjacent basal cells and course obliquely into the superficial layers where they terminate^4^. They are mainly polymodal nociceptors that are responsive to mechanical, thermal and chemical stimuli producing pain sensations^5^. There are also autonomic sympathetic nerve fibers that are derived from the superior cervical ganglia and parasympathetic fibers originated from the ciliary ganglia^1^. They function in blink reflex, tear production and secretion.

The corneal stroma is composed of predominantly collagen type I fibrils in the form of lamellae, which run orthogonally to each other giving the strength and transparency^6^. Corneal stromal keratocytes (CSKs) are a population of quiescent mesenchymal-derived cells residing between collagen lamellae^7^. The cell density is the highest in the anterior 10% of the stroma and decreases posteriorly. CSKs possess dendritic processes to connect with neighboring cells, forming a highly organized syncytium throughout the stroma. Some keratocytes are located in the vicinity of stromal nerves and occasionally enwrap nerve fibers with cytoplasmic extensions, suggesting an interdependence of the two^1^.

During LASIK (Laser-assisted *In situ* Keratomileusis), a hinged corneal flap is created, by either a microkeratome or a femtosecond laser, and the exposed stromal bed is ablated by an excimer laser^8^. The stromal nerves are transected during flap creation, and additionally through excimer ablation. The magnitude of nerve damage is related to the flap/ablation diameters and the depth and degree of laser correction^9^,^10^. The post-operative reduction of nerve fiber density in the ablation zone has been reported to be as high as 80% compared to pre-operative levels^11^. This axotomy leads to reduced corneal sensation, blink rates, lowered basal and reflex tearing and increased ocular surface exposure, that can result in LASIK-induced neurotrophic epitheliopathy. Corneal nerves can take up to 5 years to regenerate post-LASIK^11^,^12^,^13^,^14^,^15^,^16^. However, some studies have shown that corneal nerves never completely return to pre-operative levels and that the regenerated nerves have abnormal morphology (i.e. thin, greater curvature and abnormal branching)^14^,^17^,^18^.

Keratocyte loss in the stromal flap and ablation zone is commonly observed following LASIK^19^,^20^. Using *in vivo* confocal microscopy (IVCM) and histology, the CSK density has been shown to reduce and remain diminished even up to 5 years after the primary surgery^21^,^22^,^23^,^24^. Using TUNEL assay in rabbit corneas after LASIK, researchers identified that CSKs located anteriorly and posteriorly to the flap interface underwent apoptosis^25^,^26^,^27^. Several mechanisms have been considered to cause apoptosis following LASIK. Epithelial cytokines, which can enter into the stroma through a disrupted Bowman’s layer^28^,^29^ appear to be responsible for triggering apoptotic signals, and hydroxyl radical formation caused by excimer laser also induced cell death^30^.

Native CSKs are quiescent and uniquely express keratan sulfate proteoglycans (KSPGs) and stromal crystallins to aid in transparency. Upon stromal injury, the surviving CSKs transit into activated stromal fibroblasts (SFs), which are proliferative, migratory and actively producing repair-type ECM components, including fibronectin, proteinases and cell-ECM adhesion molecules (α5-integrin)^7^,^31^. They also produce chemokines that attract inflammatory cells from the limbal blood supply and tear film to the corneal stroma where these incoming cells scavenge the cellular debris^32^. Some SFs may further transform into myofibroblasts under the synergistic action of serum and TGFβ and can be associated with corneal haze [Jester 1999 JCS]^33^. Hence, CSK loss after LASIK could reduce the availability of SFs that play major roles in stromal wound healing. From a cellular point of view, wound healing in the ablation zone could have 3 main stages – acellular phase, tissue repair phase with SF repopulation and the final phase with a recovery of quiescent CSKs^34^,^35^,^36^. To date, little is known how nerves are regenerated at these stages and the expression profile of neuro-regulatory molecules.

In this study, we investigated the neuro-regulatory effect of human CSKs and SFs derived from the same donor stroma, compared to cell-free condition, using an *in vitro* chick dorsal root ganglion (DRG) model. We first validated a concentric circle intersection count (CCIC) method to quantify the extending neurites at defined distances from the DRG center. The potential secretory neuropeptides and factors that are involved in neurite growth were also identified by proteomics and Bio-Plex cytokine assay.

## Results

### Validation of concentric circle intersection counting (CCIC) method

**Figure 1:**
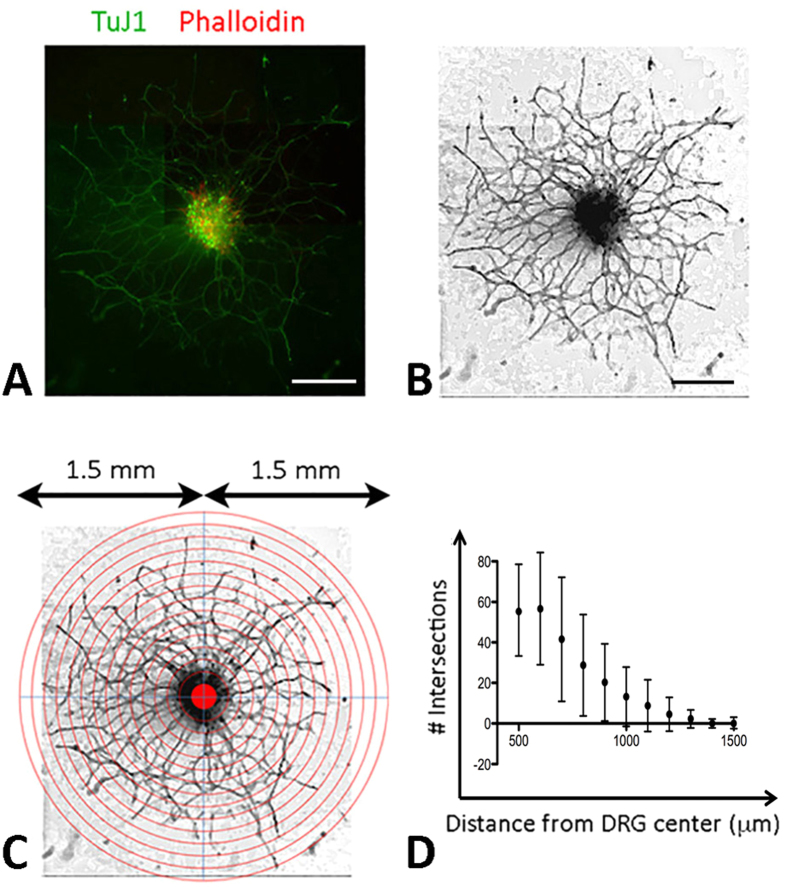
DRG neurite extension assay. (**A**) Color photographs showing representative neurite extension from DRG, (**B**) grey scale clean image and (**C**) image overlaid with concentric circles having radii increasing at 100 μm and aligned to the DRG centre. (**D**) Quantification of neurites intersecting with each circle line showed a reduction of the mean number with increasing distance from DRG center. Scale bars: 500 μm.

Using 12 DRGs incubated with medium containing 50 ng/ml nerve growth factor (NGF) for 72 hours, the immunofluorescence of TuJ1 images were converted into grey-scale and edges were enhanced (details in Methods) (Fig. 1A and B). The montaged image containing the entire neuronal network of each DRG was overlaid with a series of concentric circles with increasing radius and aligned to the DRG centre (Fig. 1C). Two masked observers manually quantified the neurites intersecting with each circle line along the distance from DRG centre. The neurite extension profile plotted with the number of intersection against distance from DRG centre showed a reduction in neurites with increasing distance from DRG center, with an average of 57.7 ± 21.1 at 500 μm to 2.3 ± 4.5 intersections at 1500 μm (Fig. 1D). The standard deviation was indicative of the variability in the intersection count ranging from 15 (DRG12) to 85 (DRG7) at 500 μm line. At longer distance from DRG center, this variability was diminished due to the reducing nerve-circle intersections. Good intra-observer agreements for each image were observed as the mean difference were <1.9 and <1.5 intersections, respectively (Table 1, Supplementary Figure 1). This represented less than 5% difference between intersection counts. Good inter-observer agreement was also achieved with a mean difference <2.8 counts (Table 1, Supplementary Figure 2). Although a statistically significant bias was seen in the count for DRG3 to 7, the Bland-Altman plots showed that LoAs were still comparatively low with a mean difference <2 for the intra-observers and <3 for inter-observers. The plots also showed that there was no evident change of LoA for intersections closer to the DRG centre, i.e. greater density of neurites. The inherent variability in the measurement of neurites in more densely packed region was deemed to be within an acceptable range.

### Cell characterization prior to conditioned media preparation

**Figure 2:**
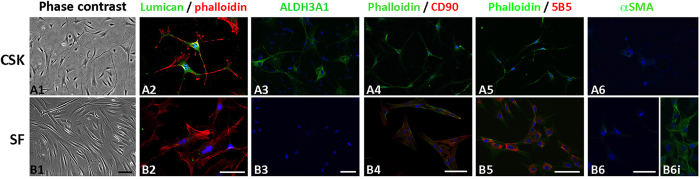
Cell characterization of CSK and SF culture from same donor. (**A** series) - CSK and (**B** series) - SF. (1) phase contrast images; (2) lumican and phalloidin staining; (3) ALDH3A1; (4) phalloidin and CD90(Thy1); (5) phalloidin and fibroblast 5B5 and (6) αSMA. (B6i) SF treated with 10 ng/ml TGFβ1 as positive αSMA staining control. Scale bars: 50 μm.

Under phase contrast microscopy, CSKs had convoluted cell body with long and slender dendritic processes forming network among cells (Fig. 2A1). They strongly expressed keratocyte markers lumican and ALDH3A1 but were devoid of fibroblast markers CD90 (Thy1), 5B5 as well as αSMA (myofibroblast marker) (Fig. 2A2 to A6). In contrast, SFs displayed long slender morphology and negligibly expressed lumican and ALDH3A1 but were positive to CD90 (Thy1) and 5B5 staining (Fig. 2B1 to B5). They were weak for αSMA, unlike SFs that were treated with TGFβ2 (10 μg/ml, positive control, Fig. 2B6 and B6i). Same marker expression pattern was also observed in CSK and SF cultures, respectively, after media conditioning, indicating that the conditioning step did not influence the cell features (Supplementary Figure 3).

### Effect of conditioned media on DRG neurite growth

**Figure 3:**
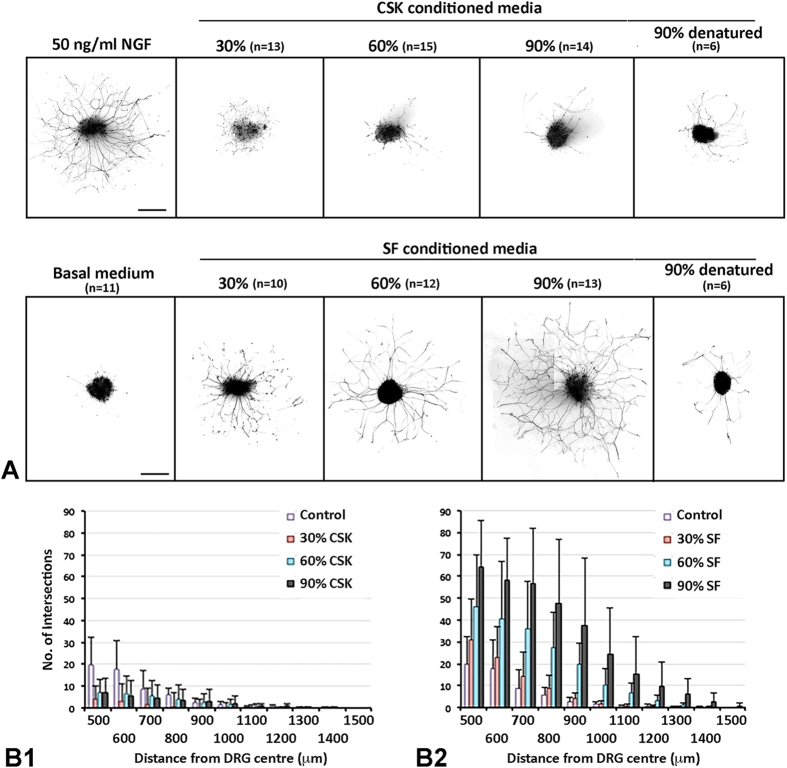
Neurite growth profile in DRG cultured with conditioned media from CSKs versus SFs of same donor. (**A**) Processed images showing representative TuJ1-stained neurite growth from DRGs cultured in different dosages of conditioned media from CSKs or SFs or in control media at 72 hours. n - number of DRG in experiment. Scale bars: 500 μm. (**B**) Histograms showing the neurite extension pattern using concentric circle intersection counting method. (**B1**) CSK conditioned media; (**B2**) SF conditioned media. Data are presented as mean and standard deviation.

The neuroregulatory effect on DRG neurite growth was evaluated after the culture of chick DRG explants for 3 days in conditioned media prepared from human CSK and SF culture, respectively, from same donor stroma. NGF was selected as the reference neurotrophin due to its high bioactivity on DRG explant cultures^37^. As depicted in Fig. 3A, a 3-day incubation of DRG explants with 50 ng/ml NGF induced significantly greater number of neurites and extension of TuJ1 positive neurite processes. In basal medium (BM) without NGF, fewer and much shorter neurites were derived from DRGs. SFCM had significantly increased bioactivity over BM control. After 3-day culture, all DRGs generated neurites over 1000 μm in length. CCIC quantification showed a dose-dependent inductive effect from 30% to 90% dosage (Fig. 3B2). Long and dense neurites were seen under higher dosage treatment. Supplementation of 90% dosage resulted in neurites reaching 1500 μm in length, whereas 30% gave neurite up to 1200 μm. Heat-denatured 90% SFCM however showed negligible effect (Fig. 3A). On the contrary, CSKCM did not show neurite induction for all 3 dosages added to DRG culture (Fig. 3A). The neurite growth patterns were indifferent to BM control. TuJ1 staining detected a few number of short neurites (predominantly in the range of 500 to 700 μm length). CCIC assay revealed a reduction of neurite density and extension when compared to SFCM (Fig. 3B1). Pairwise comparison analysis was performed using Generalized Estimating Equations (GEE) method. Supplementary Table 1 showed the independent variance structure with QICC values and estimated marginal means. Our results showed that SFCM significantly induced neurite growth when compared to BM control and a dose dependent inductive effect was likely to be observed although the difference between 60% and 90% dosages was not statistically significant (Table 2). For CSKCM, all 3 dosages showed insignificant effect on neurite growth.

### Protein profiling in CSK and SF conditioned media

**Figure 4:**
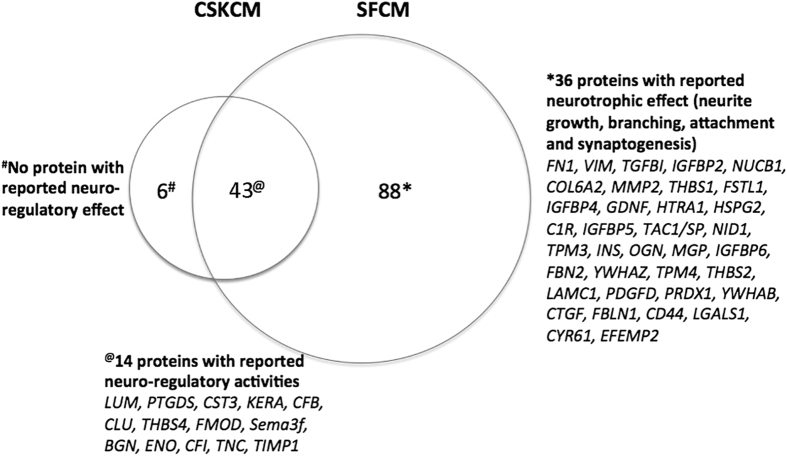
A scaled Venn diagram illustrating the protein composition in conditioned media (CM) from human CSKs and SFs identified by nanoLC-MS/MS analysis. Proteins with reported neuro-regulatory functions were listed.

Using nanoLC-MS/MS analysis and ProteinPilot search against InterPro human protein database, a total of 49 proteins from CSKCM and 131 proteins from SFCM samples were successfully mapped with peptide homology ≥95% (Supplementary Table 2). Forty-three proteins were commonly expressed in both samples, accounting for 88% in CSKCM and 33% in SFCM. Out of 88 proteins found unique to SFCM, 33 had reported neuro-regulatory activities (protein list in Fig. 4). This was not found for CSKCM-only proteins (i.e. KRT14, ITIH4, MFI2, SAMD15, LIG4, CFH). Using web-based DAVID for enriched GO terms, both CSKCM and SFCM samples showed the prediction for ECM, ECM-receptor interaction and structural constituents of cytoskeleton (Table 3). Additional enriched GO terms including focal adhesion (*P* = 4 × 10^−10^), response to wounding (*P* = 2 × 10^−9^) and regulation of neurogenesis (*P* = 3 × 10^−5^) were exclusive to SFCM, while complement activation (*P* = 2 × 10^−7^), iron transport (*P* = 1.4 × 10^−3^) and glycosaminoglycan metabolism (*P* = 1.4 × 10^−2^) were found for CSKCM.

### Comparison of cytokine/chemokine/growth factor profiles in conditioned media from stromal fibroblast and corneal stromal keratocytes

**Figure 5:**
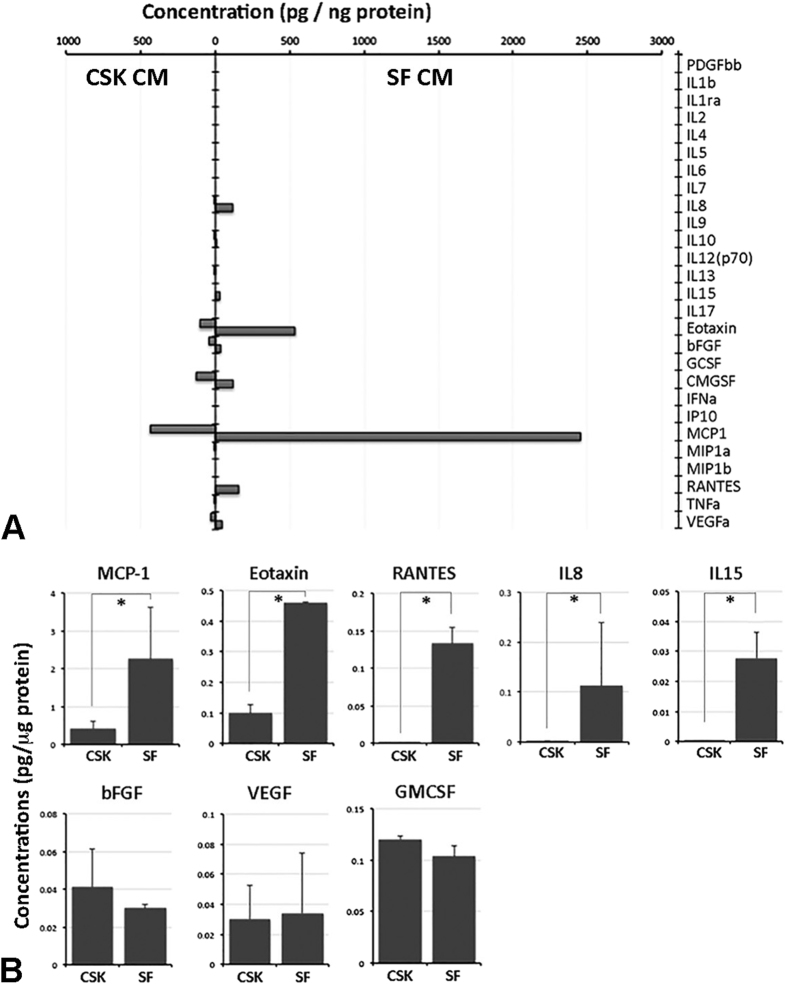
Identification of cytokine, chemokines and growth factors in conditioned media (CM) from CSK and SF cultures by Bioplex 27-plex assay. (**A**) Histogram showing the concentrations (pg/ng protein) of protein candidates in either CM samples. (**B**) Graphs comparing the concentrations of 8 detected factors in SFCM versus CSKCM by Bioplex assay. Data are presented as mean and standard deviation. *P < 0.05, Mann-Whitney U test.

Bio-Plex Human Cytokine 27-plex panel was employed to measure the concentrations of 27 human cytokines, chemokines and growth factors in CM samples in duplicated runs. Out of 27 candidates, 8 factors were detectable accounting for a detection rate of 30% (Fig. 5A). Figure 5B showed the mean concentrations with standard deviation. The concentrations of IL8 (*P* = 0.004, Mann-Whitney U test), IL15 (*P* < 0.01), MCP-1(CCL2) (*P* = 0.01), Eotaxin(CCL11) (*P* < 0.01) and RANTES(CCL5) (*P* < 0.01) in SFCM were significantly higher than in CSKCM. In contrast, bFGF, VEGF and GM-CSF had no significant difference in the concentrations between the two conditioned media.

### Cellular expression of candidate neuroregulatory genes in human stromal fibroblast and corneal stromal keratocytes

**Figure 6:**
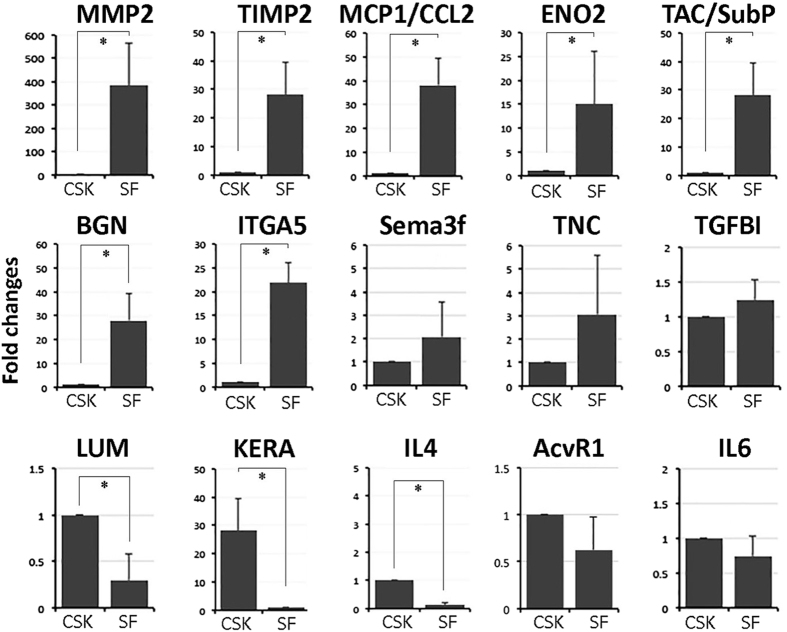
Quantitative PCR analysis of neurite growth-associated gene expression in CSKs and SFs from same donor. Graphs showed the fold changes of target gene expression in human SF versus that of CSK from same donors. Data are presented as mean and standard deviation. *P < 0.05, Mann-Whitney U test.

We examined candidate gene expression in human CSK and SF by quantitative PCR (Fig. 6). SF significantly expressed MMP2 (fold change 383 ± 182; *P* < 0.01, Mann-Whitney U test), TIMP1 (fold change 28 ± 11; *P* < 0.02), MCP1/CCL2 (fold change 38 ± 11; *P* < 0.01), enolase ENO2 (fold change 15 ± 11; *P* < 0.05), tachykinin precursor 1 (TAC1)/substance P (SP) (fold change 30 ± 12; *P* < 0.01), biglycan BGN (fold change 28 ± 12; *P* < 0.02) and ITGA5 (fold change 22 ± 4.2; *P* = 0.02) when compared to CSK. The expression of Sema3f, TNC and TGFBI was higher in SF but did not reach the statistical significance. In contrast, CSK expressed significantly higher levels of lumican LUM (fold change 4 ± 0.8, *P* < 0.05), keratocan KERA (fold change 28 ± 11, *P* < 0.02) and IL4 (fold change 11 ± 3.6, *P* < 0.02).

## Discussion

DRG explant culture is a common model of identifying neurite growth activity, however the response can be difficult to be quantified. Various studies quantifying nerve growth have employed manual counting, outgrowth by distance and semi-automated and automated nerve quantification software, e.g. NeuronJ^38^,^39^. More quantitatively, the measurement of neurite growth area, density, branch points and selective measurement of neurite length can be undertaken^38^,^40^,^41^. However, there exist problems associated with neurite fasciculation and branching. Also, neurite counting fails to account for differences of neurite length. Concentric circle intersection count (CCIC) method has been used to quantify the extending neurites at different distances from a defined origin^39^,^42^. However, no method validation has been reported and the accuracy of measurement can be influenced by the reproducibility of counting done by different observers. Hence, we first performed a validation process to evaluate the reproducibility and repeatability of CCIC by masked observers. Our results have shown that this assay is a highly reproducible tool for quantitative measurement of neurite growth. Tested with 12 DRGs treated with 50 ng/ml NGF, the mean of differences between measurements by the same observer was small along the entire distance range (500 to 1500 μm). The means of difference between measurements by different observers were also small. LoAs were low for both inter- and intra-observer variation. Minor variations were detected which might be due to TuJ1 immunostaining of which some neurites had weak to almost undetectable signal that might not be distinguishable by some observers. Interestingly, several P values were statistically significant for counting by observer 1 (DRG3 to 7), however, most P values for observer 2 were found to be >0.05. This led to the overall LoA to be comparatively low with a mean difference <2 for intra-observers and <3 for inter-observers. To assess if the outcome of CCIC method can be comparable to other independent methods, we additionally performed total neurite density measurement using NeuronJ (ImageJ, National Institute of Health, US) and Quantity One analysis (BioRad). For 5 randomly selected DRGs, we observed that the total neurite density was highly correlated with the CCIC profile, of which the Area of Under Curve (AUC) was calculated (Supplementary Figure 4), suggesting that CCIC can be a valid method for the analysis of neurite outgrowth from a neural tissue.

We then studied the neurotrophic effect of human CSKs and SFs using a chick DRG model. SF secretion significantly induced neurite outgrowth and extension compared to CSK and the effect was dose-dependent. Using nanoLC-MS/MS and Bio-Plex cytokine assays, we detected an array of neurotrophic factors uniquely secreted by SFs. Enriched gene ontology term analysis predicted the regulation of neurogenesis, focal adhesion and wound healing exclusively for SF secreted proteins. In contrast, CSK secretion and basal medium (cell-free) control had negligible influence on neurite growth. CSKs secreted much fewer proteins with reported neuro-regulatory activity and these molecules were also secreted from SFs. This differential neurotrophic response caused by human SFs and CSKs was not due to donor variation as both cell types were derived from the same stroma to ensure same cell source identity. The cells were characterized and expressed phenotypic markers for CSK (lumican, ALDH3A1) and SF (Thy1, fibroblast 5B5), respectively, and the absence of αSMA, indicating no transition to myofibroblasts. There were no changes of cell features due to media conditioning step as similar marker expression pattern was observed in CSK and SF cultures, respectively. The serum effect (in SF culture) was removed by extensive washes and an overnight weaning before medium conditioning. Hence, the neurite growth profile was exclusively caused by factors secreted from human CSKs or SFs. Also, the neuro-regulatory effect was abolished after thermal denaturation of conditioned media samples, indicating the biological specificity.

The dose-dependent neurotrophic effect shown by the SFs has suggested that neurite growth could be affected by the number of SFs. Immediately after LASIK photoablation, CSK loss in the stromal flap and retroablation zone could diminish the viable CSK pool which is the source of SF conversion^24^,^25^,^26^,^30^,^43^. The acellular zone requires repopulation by migration of SFs derived from uninjured CSKs outside the ablation zone or from the stroma anterior to the interface. In a clinical study, the activation of CSKs located in the stromal bed was detected from the third post-operative day^26^. By IVCM, these activated cells are visualized by the brightly reflective oval nuclei, slowly migrated to repopulate the acellular region^34^. However, hypocellularity in the anterior flap region was still significant at 6 months to 2 years post-LASIK and the cell density may only reach 60% as that of the pre-operative state^26^. This indicates that a prolonged period of time is required to repopulate SFs for sufficient stimulation to nerve regrowth. The process can take as long as 5 years to complete^11^,^12^,^13^,^14^,^35^,^44^.

Recently, SMILE (SMall Incision Lenticule Extraction), an all-in-one femtosecond procedure by Zeiss, was approved by The Food and Drug Administration (FDA), US (http://www.meditec.zeiss.com/press). The creation of a flap is obviated, instead a piece of lenticule is dissected and extracted from a small arcuate incision (2–4 mm)^16^,^45^. Unlike LASIK, SMILE lenticule dissection employs a femtosecond laser at near-infrared wavelength range to photo-disrupt stromal tissue with high precision and less thermal damage^46^,^47^. Our previous study showed that SMILE induced less CSK apoptosis and inflammation compared to LASIK, thus creating a much thinner acellular zone^46^. The CSKs remained viable and could repopulate early along the femtosecond laser incision site and this appeared to occur through the migration of adjacent keratocytes^48^,^49^. SMILE has also been shown to damage less stromal nerves, preserve the sub-basal nerve plexus and have faster nerve recovery compare to LASIK^16^,^50^. In this study, we have shown that nerve growth was SFCM dose-dependent due to the presence of neurotrophic factors and cytokines. A constitutive cellular secretion from cultured fibroblasts has been reported to be cell density-dependent^51^. Hence, more SFs that can be promptly generated from the viable CSKs at the interface region will produce greater neurotrophic effect via its secretion and result in more efficient nerve regeneration.

Our proteomics study showed that CSK and SF not surprisingly had different secreted protein profiles (Fig. 4). CSK released fewer proteins than SF and 43 out of 49 proteins (88%) were also found in SF secretion. Over 80 proteins were uniquely present in SF secretion. Examining the top 30 proteins in both categories, all CSK-secreted proteins have been verified^52^,^53^. Not surprisingly, it contained proteins related to ocular transparency (including LUM, KERA, SPARC), stromal deposition and immune response (including CLU, ALB, CFB, C1S, APOD, THBS4). In the SF secretion, most were also previously verified, including those for fibrogenesis and wound healing response (FN1, COL5A2, VIM, TGFBI, IGFBP2, IGFBP4, COL6A2, MMP2, HTRA1), and immune response (HTRA1, NID, TAC1, C1R, PTX3). However, nucleobindin-1 (NUCB1) is reported for the first time in association with corneal stroma. It is a calcium-binding Golgi protein of neuron and can regulate cell survival against unfolded protein response stress^54^ and immune metabolic interaction^55^. Besides, the expression of CLAU and CLSTN1 may involve in Ca^++^ regulation for transparency^52^,^56^. Using multiplex human cytokine assay, we also identified for the first time IL15 in SF secretion.

More than one-third of SF-secreted proteins had known inductive effects on nerve regeneration, sprouting, growth and differentiation (Fig. 4). For instance, substance P (SP), acts as neuro-transmitter and modulator for pain sensitization and neurogenic inflammation. In the cornea, SP is not only detectable in nerve fibers, but also in corneal epithelial and stromal cells^57^,^58^. SFs also secreted IL8 and other cytokines, including IL15, MCP-1/CCL2 and RANTES/CCL5, which were absent from CSK’s, both under condition without any stimulating factors. Together with IL15, these pro-inflammatory molecules are important for neutrophil recruitment and T-cell activation^59^,^60^. They are also reported as neurotrophins: IL8 and 15 for neural cell differentiation via STAT3 pathway^61^, MCP-1 for neuron function and sensitivity^62^ and RANTES for neuron survival and differentiation including synaptogenesis^63^. Their production in SFs indicates that nerve regeneration and inflammatory events could share common pathways in tissue repair processes. Nerve injury inevitably initiates inflammation that involves multiple events to recruit immune cells. Hence, inflammation management could be a strategy for neuro-regeneration^64^.

Other SF secreted proteins can be categorized into ECM proteins associated with neurite initiation, growth and elongation, topographic guidance and protection from apoptosis, including FN, LAM, CTGF, CYR61, COL6A2, HSPG2, BGN, MGP and TNC^65^,^66^,^67^,^68^. Moreover, matricellular proteins like MMP2, TIMP, GDNF, PDGF, IGFBPs, THBS and complement factor C1R have been reported to promote neurite extension, adhesion, branching and differentiation as well as protection from neurotoxicity^69^,^70^,^71^,^72^. Also found unique in SF secretion, PRDX1, coding for antioxidant enzyme, has been reported in neuroprotection against oxidative stress^73^. The semaphorin (Sema) family contains short-range diffusible members that primarily work as axon guidance, hence they are important in neuron development and also in immune functions^74^. Sema7a was expressed in regenerating murine nerve spouts after corneal lamellar corneal surgery^75^. Our study, however, identified greater Sema3f expression in human SFs than CSKs, which could be due to axon-related expression^76^. Taken together, SFs released neurotrophins, immune regulatory factors and matricellular proteins to promote nerve regeneration, growth, differentiation and wound healing. Further investigation on these peptides/growth factors will give a better understanding about corneal nerve regeneration, stromal wound healing and pain management following laser-associated refractive surgeries.

This is the first study showing the physiologic relationship between nerve and stromal fibroblasts versus stromal keratocytes from the same cornea source. Our results demonstrated that human SFs exerted significantly greater neurotrophic activities than the quiescent CSKs. Hence the early repopulation of SFs after refractive surgery could promote nerve regeneration. In comparison to LASIK, SMILE induces less CSK death, less nerve damage and thinner acellular zone than LASIK. The presence of more viable CSKs will result in a prompt activation to SFs at the interface site, hence offers an explanation for possibly inducing faster nerve regeneration^16^.

## Methods

### Chick dorsal root ganglion (DRG) isolation and culture

Chick embryos (embryonic day E9 to 10) were sacrificed by decapitation. The visceral organs and tissues were removed to expose the lumbar DRGs, which were collected in ice-cold phosphate buffered saline (PBS, 0.01 M, First Base). DRGs were cleaned and peripheral tissue was carefully trimmed. Each DRG was bisected to allow efficient outgrowth of neurites. Each half of DRG was placed on collagen I-coated sterile coverslips with the bisected surface facing to the substrate. For the validation study, DRGs were cultured in DMEM/F12 medium (Invitrogen, Life Technologies) added with 5% heat-denatured foetal bovine serum (FBS, Invitrogen), nerve growth factor (NGF 2.5S, 50 ng/ml, Invitrogen) and antibiotics (penicillin S, streptomycin sulfate and amphotericin B, Invitrogen) for 72 hours before fixation for TuJ1 immunostaining. For co-culture experiment, the DRGs were then incubated in culture media prepared with the addition of 10%, 20% and 30% conditioned medium (CM) concentrate (vol/vol) from either CSK or SF culture in serum-free basal medium (refer to subsequent methods).

### Primary cultures of human corneal stromal keratocytes and stromal fibroblasts

**Figure 7:**
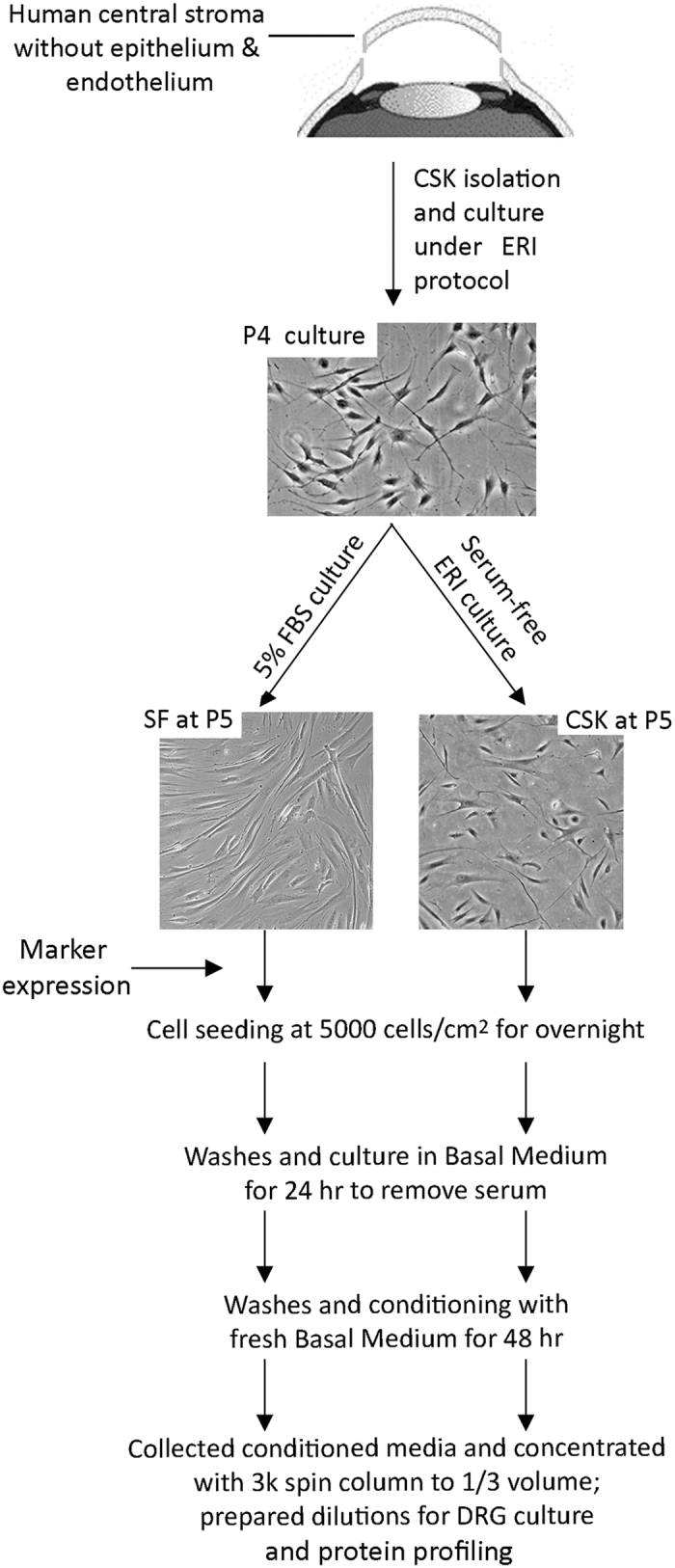
Schematic diagram showing CSK and SF culture from same donor stroma and preparation of conditioned media.

Six research grade cadaveric corneal tissues (information provided in Supplementary Table 3) were purchased from Lions Eye Institute for Transplant and Research Inc. (Tampa, FL, USA) following institutional review board approval, in accordance with approved guidelines. Consent was taken at the time of retrieval by next of kin for use for research and the protocol was authorized by Lions Eye Institute, as the human tissue was deemed unsuitable for transplantation. Corneal tissues were transported in Optisol-GS (Bausch & Lomb Surgical, Irvine, CA, USA) at 4 **°**C to the laboratory. Donor information is listed in Supplementary Table 1. The central button (8 mm in diameter) was trephined and treated with dispase II (20 mg/ml, Roche, Basal, Switzerland) to completely remove corneal epithelium and endothelium (Fig. 7). The stromal tissue fragments were digested with collagenase I (1 mg/ml, Worthington, Lakewood, NJ, USA) for 6 to 8 hours at 37 °C. Single cells were suspended in 0.5SERI medium^77^, which was DMEM/F12 containing 1 mM L-ascorbate 2-phosphate (Sigma, St Louis, MO, USA), soluble amnion stromal extract (ASE, 5 μg protein/ml), ROCK inhibitor Y27632 (10 μM, Millipore, Billerica, MA, USA), insulin-like growth factor 1 (IGF1, 10 ng/ml, Invitrogen), 0.5% FBS and antibiotics. Cells were seeded at 10^4^ cells/cm^2^ on collagen I-coated glass surface (BD Biosciences, Franklin Lakes, NJ, USA). Fresh medium was replenished every 3 days. When “activated keratocytes” reached about 70% confluence, they were dissociated with TryPLE Express (Invitrogen) and plated on new collagen I-coated surface under the same seeding density. At passage 4, one half of activated keratocytes were changed to serum-free ERI culture for the reversal to CSKs and the other was cultured with 5% FBS to convert into SFs. This step was continued for 7 days with fresh media replenished every 2 days. Both cell types were characterized by marker expression prior to conditioned media preparation.

### Conditioned media preparation, dilutions and treatment of chick DRG

Human CSKs and SFs from same donor stroma were seeded at 5000 cells/cm^2^ on collagen I-coated culture surface in respective culture media. After overnight, the attached cells were washed twice with sterile PBS and then with serum-free basal medium (BM) (DMEM/F12 added with 2 mM glutamine, 1 mM ascorbate 2-phosphate and antibiotics), which was discarded after 24 hours (Fig. 1). This eliminated serum or growth supplements from original cultures. Following this, fresh serum-free BM (0.2 ml/cm^2^ surface) was added and incubated for 48 hours. The conditioned media (CM) from either CSKs or SFs were collected and spun at 300 g for 5 min to remove cellular debris. Clear CM supernatants were concentrated using Amicon Ultra-15 Centrifugal Filter Unit (Millipore) at 14000 rpm for 8 min to 1/3 of initial volume. The CM concentrates were added as 10%, 20% and 30% in BM (vol/vol) for DRG co-culture. With a concentration factor of 3, the reconstituted media contained 30%, 60% and 90% of initial CM. Negative control was BM only and positive control was BM added with 50 ng/ml NGF. After 72 hours, DRGs and neurite explants were fixed *in situ* with 2% neutral buffered paraformaldehyde (Sigma).

### Immunofluorescence

After fixation, the samples were quenched with ice-cold 50 mM ammonium chloride (Sigma), permeabilized with 0.15% saponin (Sigma) and blocked with 1% bovine serum albumin (BSA, Sigma) and 2% normal goat serum (Invitrogen), the samples were incubated with primary antibodies (Supplementary Table 4) for 2 hours at room temperature. After washing with PBS, they were labelled with Red-X or Alexa488-conjugated IgG secondary antibody (Jackson ImmunoRes Lab, West Grove, PA, US) and fluorescein-conjugated phalloidin. After washes, samples were mounted with Fluoroshield with DAPI (4′,6-diamidino-2-phenylindole; Santa Cruz Biotech) and viewed under fluorescence microscopy (Carl Zeiss, Oberkochen, Germany).

### Growth quantification of TuJ1 expressing neurite by concentric circle intersection count (CCIC) and method validation

The captured TuJ1 fluorescence images were converted into grey-scale at resolution of 300 pixels per inch using Photoshop CC (Adobe) and edge enhancement with a sobel filter and HDR toning at 0.5 strength. The background non-neural cells were eliminated through colour value segmentation. The final images were montaged to portray the entire neuronal network of each DRG (Fig. 1A and B). A series of concentric circles with radius increasing at 100 μm and spanning from 500 to 1500 μm range were overlaid on the final image and aligned to the DRG centre, which was distinguished by the intersection of 2 longest diagonal lines inside DRG block (Fig. 1C). The number of neurites intersecting with each circle line along the distance from DRG centre was then manually quantified. The neurite extension profile was plotted with the number of intersection against distance from DRG centre (Fig. 1D). For method validation, two masked observers performed the same quantification procedure using a set of 12 DRG images. The exercise was repeated after 48 hours and we compared the inter-observer agreement and intra-observer reproducibility.

Quantification data from experiments was analyzed by pairwise comparison using Generalized Estimating Equations (GEE) method to calculate longitudinal correlated data (i.e. distance repeated measures). Intersection count data were fitted to a Poisson regression model giving the distribution with a clustering on zero as the count approaching more distance measurements. The corrected quasi-likelihood under independence model criterion (QICC) values were used to compare different CM dosages (the lower QICC the better model fitting). Independent, unstructured and exchangeable variance structures were compared using QICC values.

### One-dimensional nano-scale liquid chromatography coupled to tandem mass spectrometry (nanoLC-MS/MS) and pathway analysis

Sample preparation, MS and data collection followed the standard protocols described in our previous study^77^. In brief, protein sample (50 μg) dissolved in 50 mM ammonium bicarbonate (Sigma) and 5 mM TCEP, followed by washes with 75% urea/Tris. Iodoacetamide (Sigma) was added to a final concentration of 15 mM to alkylate the reduced proteins. After urea washes, sample was trypsinized in 50 mM triethylammonium bicarbonate (Sigma) for 16 hrs. The digested sample was reconstituted in 0.1% formic acid, 2% acetonitrile and analyzed by one-dimensional nanoLC-MS/MS (Dionex Ultimate 3000; ThermoFisher Scientific, Waltham, MA, US) coupled with Triple TOF 5600 (AB SCIEX, Framingham, MA, US). MS/MS data output was analyzed by ProteinPilot 5.0 (AB SCIEX) with a search against Swiss Prot_Human_June2016_Reviewed version to identify proteins based on False Discovery Rate (FDR) set at <1%. Gene Ontology analysis was done by input protein list to web-based DAVID (Database for Annotation, Visualization and Integrated Discovery) V6.8 (https://david.ncifcrf.gov/home.jsp). *P* < 10^−4^ indicated the significantly enriched GO terms.

### Cytokine measurement analysis

The expression of cytokines, chemokines and growth factors in conditioned media was profiled by multiplex bead assay instead of MS due to the sensitivity and protein degradation issue during sample processing. The experiment was performed using Bioplex Pro (27-plex) assay (BioRad). The protein list included: (A) growth factors: basic fibroblast growth factor (bFGF), vascular endothelial growth factor (VEGF), granulocyte colony stimulating factor (G-CSF), platelet-derived growth factor bb (PDGF-bb); (B) cytokines: interleukin (IL)-1Ra, IL-1b, IL-2, IL-4, IL-5, IL-6, IL-7, IL-8, IL-9, IL-10, IL-12, IL-13, IL-15, IL-17, interferon-γ (IFNγ), granulocyte-macrophage colony stimulating factor (GM-CSF), tumor necrosis factor-α (TNFα) and (C) chemokines: macrophage inflammatory protein (MIP)-1a, 1b, monocyte chemoattractant protein-1 (MCP-1), eotaxin-1 (CCL11), interferon inducible protein-10 (IP-10), regulated upon activation, normal T cell expressed and presumably secreted (RANTES).

The assay was conducted according to manufacturer’s instruction. In brief, a standard curve was created ranging from 1.95 to 32,000 pg/ml. In a 96-well filtration plate, premixed beads coated with target antibodies was added, and washed twice with Bio-Plex wash buffer. Pre-mixed standards or samples were then added, mixed and incubated for 30 min with shaking at 300 rpm at room temperature. After buffer washes, streptavidin-phycoerythrin (PE) was added and incubated for 10 min with shaking. After washes, Bio-Plex assay buffer was added to beads and samples were measured using Bio-Plex suspension array system and data analyzed using Bio-Plex Manager software with 5PL curve fitting.

### Quantitative PCR analysis

Cells were collected in RLT buffer and RNA was extracted using RNeasy kit (Qiagen, Valencia, CA, US) and on-column RNase-free DNase kit (Qiagen) according to manufacturer’s protocol. In brief, total RNA (1 μg) was reverse transcribed using Superscript III RT-PCR kit (Invitrogen) with random hexanucleotide primer (10 ng/ml, Invitrogen). Gene expression was assayed with specific primer pairs (Supplementary Table 5) by quantitative real-time PCR (qPCR) using Sybr Green Supermix (BioRad) in GFX96 Real-time System (BioRad). Experiments were run in quadruplicate. Relative gene expression of each sample (ΔCT) was normalized by the mean CT of the housekeeping gene glyceraldehyde-3-phosphate dehydrogenase (GAPDH) (CT_GAPDH_) and expressed as mean and standard deviation (SD). Fold changes comparing expression in SF versus CSK were calculated.

### Statistical analysis

In the CCIC validation study, the agreement was calculated by using Bland-Altman plots with mean difference and level of agreement (LoA) and paired t test together with Prism 5.0 for Macintosh as previously described^38^,^78^. For the DRG neurite growth study in conditioned media, at least 3 biological replicates were performed. Each culture condition was analyzed for 6 DRG explants in each biological replicate. Pairwise comparison of neurite intersection count among treatments was performed using Generalized Estimating Equations (GEE) method and data fitting to Poisson regression model. Mann-Whitney U test was used to compare the ranked neurite length data between treatment and control and qPCR gene expression levels. Bioplex analysis was performed in duplicate. Results were described as a mean and SD. All statistical calculation was performed using SPSS 20.0 (SPSS, Chicago, IL, US). Means were compared using one-way ANOVA after data were log transformed to improve normality. For all assays, *P* < 0.05 was considered statistically significant.

## Additional Information

**How to cite this article**: Yam, G. H.-F. *et al*. Nerve regeneration by human corneal stromal keratocytes and stromal fibroblasts. *Sci. Rep.* **7**, 45396; doi: 10.1038/srep45396 (2017).

**Publisher's note:** Springer Nature remains neutral with regard to jurisdictional claims in published maps and institutional affiliations.

## Supplementary Material

Supplementary Materials

## Figures and Tables

**Table 1:** Intra- and inter-observer variability of CCIC method analyzed by Bland-Altman plots with mean difference and level of agreement (LoA).

DRG	Observer 1	Observer 2	P	Mean bias (95% CI)
	Mean (SD)	Mean (SD)	(Two-sided)	
***Inter-observer variability***				
1	12.18 (12.21)	11.82 (11.97)	0.371	0.36 (−2.157 to 2.885)
2	7.091 (6.534)	7.182 (6.615)	0.67	−0.09 (−1.46 to 1.28)
3	31.09 (7.867)	28.82 (7.167)	**<0.001**	2.27 (0.11 to 4.43)
4	30.82 (24.71)	29.64 (23.75)	**0.01**	1.18 (−1.27 to 3.63)
5	58.00 (38.60)	55.36 (37.27)	**0.01**	2.63 (−1.77 to 7.04);
6	46.18 (17.39)	43.73 (15.82)	**0.02**	2.46 (−3.05 to 7.95)
7	50.09 (37.43)	47.27 (34.94)	**<0.01**	2.82 (−2.50 to 8.14)
8	11.45 (12.32)	10.82 (10.59)	0.36	0.64 (−3.68 to 4.96)
9	12.73 (18.52)	12.64 (17.99)	0.80	0.09 (−2.14 to 2.32)
10	31.36 (22.82)	31.36 (22.82)	1.0	0.00 (−2.32 to 2.32)
11	23.00 (27.01)	21.36 (25.55)	**0.01**	1.64 (−1.68 to 4.95)
12	9.909 (7.231)	9.727 (6.944)	0.62	0.18 (−2.11 to 2.47)
***Intra-observer 1 variability***				
**DRG**	**First Analysis**	**Second Analysis**	**P**	**Mean bias (95% CI)**
	**Mean (SD)**	**Mean (SD)**	**(Two-sided)**	
1	12.18 (12.21)	12.18 (12.15)	1.0	0.00 (−1.24 to 1.24)
2	7.091 (6.534)	6.909 (6.457)	0.17	0.18 (−0.61 to 0.97
3	31.09 (7.867)	30.27 (6.987)	**0.04**	0.82 (−1.47 to 3.11)
4	30.82 (24.71)	30.0 (24.12)	**0.03**	0.82 (−1.30 to 2.93)
5	58.00 (38.60)	57.27 (37.80)	**0.02**	0.73 (−2.56 to 4.02)
6	46.18 (17.39)	44.27 (16.40)	**0.01**	1.91 (−1.65 to 5.47)
7	50.09 (37.43)	48.36 (36.66)	**0.04**	1.73 (−2.84 to 6.29)
8	11.45 (12.32)	11.00 (11.78)	0.10	0.45 (−1.15 to 2.06)
9	12.73 (18.52)	12.18 (17.09)	0.34	0.55 (−3.00 to 4.09)
10	31.36 (22.82)	31.64 (23.05)	0.57	−0.27 (−3.30 to 2.78)
11	23.00 (27.01)	22.27 (26.35)	0.07	0.73 (−1.61 to 3.06)
12	9.909 (7.231)	9.727 (6.944)	0.34	0.18 (−1.00 to 1.36)
***Intra-observer 2 variability***				
**DRG**	**First Analysis**	**Second Analysis**	**P**	**Mean bias (95% CI)**
	**Mean (SD)**	**Mean (SD)**	**(Two-sided)**	
1	11.82 (11.97)	11.64 (12.02)	0.5	0.18 (−1.53 to 1.90)
2	7.182 (6.615)	7.091 (6.441)	0.77	0.09 (−2.14 to 2.31)
3	28.82 (7.167)	27.36 (7.366)	**0.04**	1.46 (−2.60 to 5.50)
4	29.64 (23.75)	29.09 (23.41)	0.11	0.55 (−1.49 to 2.58)
5	55.36 (37.27)	54.91 (36.50)	0.48	0.45 (−3.60 to 4.51)
6	43.73 (15.82)	43.36 (15.81)	0.44	0.36 (−2.58 to 3.30)
7	47.27 (34.94)	46.36 (34.21)	0.13	0.91 (−2.65 to 4.47)
8	10.82 (10.59)	10.64 (11.08)	0.5	0.18 (−1.53 to 1.90)
9	12.64 (17.99)	12.55 (17.87)	0.72	0.09 (−1.54 to 1.72)
10	31.36 (22.82)	30.55 (21.83)	0.15	0.82 (−2.56 to 4.19)
11	21.36 (25.55)	20.73 (24.19)	0.39	0.64 (−3.94 to 5.20)
12	9.727 (6.944)	9.818 (7.097)	0.76	−0.09 (−1.94 to 1.76)

Note: P values were calculated by 2-sided paired t-test. Limits of agreement were set as mean difference ±1.96 × SD. Upper and lower limits of agreement indicate 95% confidence range.

**Table 2:** Pairwise comparison analysis (P values) of neurotrophic effect of SF and CSK conditioned media.

		Basal media	SFCM			CSKCM		
			30%	60%	90%	30%	60%	90%
	**Basal media**		**0.012**	**<0.001**	**<0.001**	0.131	0.284	0.510
SF CM	**30%**	**0.012**		0.033	**0.001**	**<0.001**	**<0.001**	**0.008**
	**60%**	**<0.001**	0.033		0.262	**<0.001**	**<0.001**	**<0.001**
	**90%**	**<0.001**	**0.001**	0.262		**<0.001**	**<0.001**	**<0.001**
CSK CM	**30%**	0.131	**<0.001**	**<0.001**	**<0.001**		0.754	**0.014**
	**60%**	0.284	**<0.001**	**<0.001**	**<0.001**	0.754		0.238
	**90%**	0.510	**0.008**	**<0.001**	**<0.001**	0.114	0.238	

*P* values of pairwise comparisons of estimated margin means for DRG treatment with CSK, SF conditioned media and basal media. *P* < 0.05 was statistically significance.

**Table 3:** Enriched Gene Ontology (GO) terms predicted for CSKCM and SFCM proteins (top 10 candidates).

Rank	CSKCM		SFCM	
	Enriched GO Term	*P*^#^	Enriched GO Term	P^#^
1	Extracellular region	**3 × 10**^**−20**^	Extracellular region	**2 × 10**^**−43**^
2	Complement activation	**2 × 10**^**−7**^	ECM-receptor interaction	**2 × 10**^**−15**^
3	ECM-receptor interaction	**3 × 10**^**−6**^	Growth factor binding	**3 × 10**^**−12**^
4	Cytoskeletal constituents	**6 × 10**^**−5**^	Focal adhesion	**4 × 10**^**−10**^
5	Endopeptidase activity	**6 × 10**^**−5**^	Response to wounding	**2 × 10**^**−9**^
6	Cytoplasmic vesicles	6.7 × 10^−3^	Polysaccharide binding	**5 × 10**^**−9**^
7	Iron transport	1.4 × 10^−3^	Ectoderm development	**8 × 10**^**−7**^
8	Glycosaminoglycan metabolism	1.4 × 10^−2^	Cytoplasmic vesicles	**1 × 10**^**−5**^
9	Calcium binding	2.5 × 10^−2^	Regulation of neurogenesis	**3 × 10**^**−5**^
10	Regulation of cell proliferation	4.1 × 10^−2^	Cytoskeletal constituents	**3 × 10**^**−5**^

^*#*^*P* values were calculated by Fishers Exact T test associated with the biological process.

Bold - significant enriched GO terms with *P* < 10^−4^.
