# Supplementary material for: Nerve regeneration by human corneal stromal keratocytes and stromal fibroblasts
Source: Sci Rep. 2017 Mar 28;7:45396. doi: 10.1038/srep45396 (PMC5369053; doi:10.1038/srep45396)
Supplement: Supplementary Materials [file srep45396-s1.pdf]

Nerve regeneration by human corneal stromal keratocytes and stromal fibroblasts

Gary Hin-Fai Yam, Geraint P Williams, Melina Setiawan, Nur Zahirah Binte M Yusoff, Xiao-wen Lee, Hla Myint Htoon, Lei Zhou, Matthias Fuest, Jodhbir S Mehta

Supplementary Materials

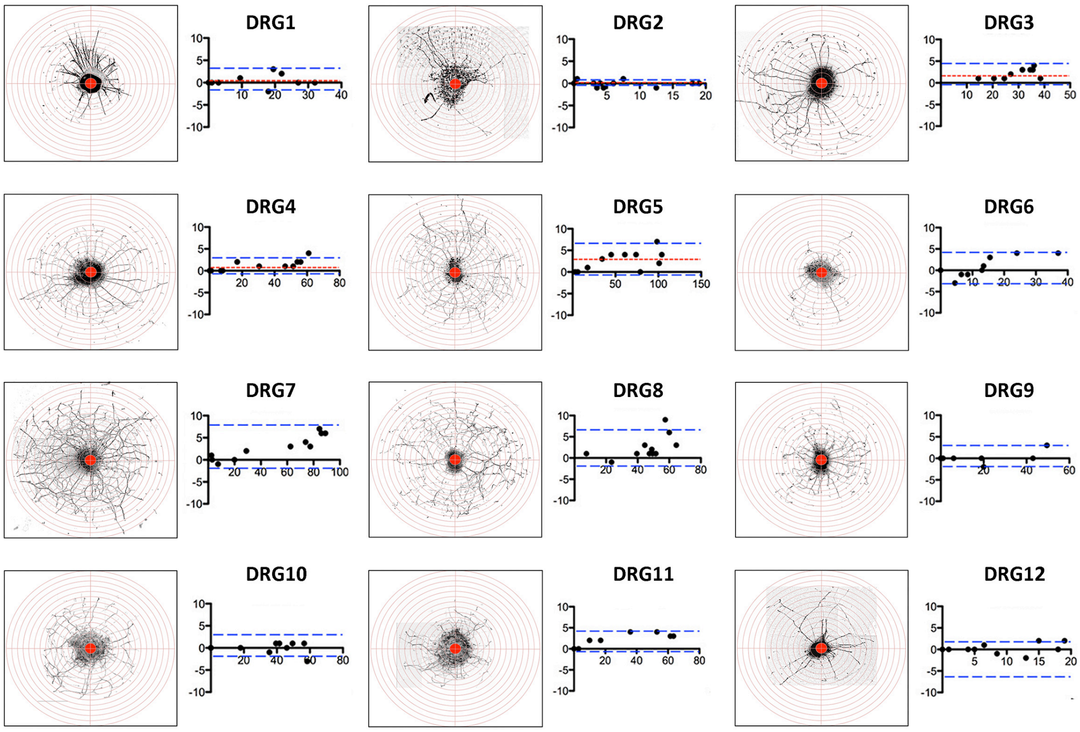

Supplementary Figure 1. Color photographs of neurite extension with concentric circle overlay for validation study (12 samples). Bland-Altman plots demonstrated inter-observer agreement. The upper and lower 95% limits of agreement from the mean difference (red hatched lines) were shown (defined as +/-1.96 SD [blue hatched lines]).

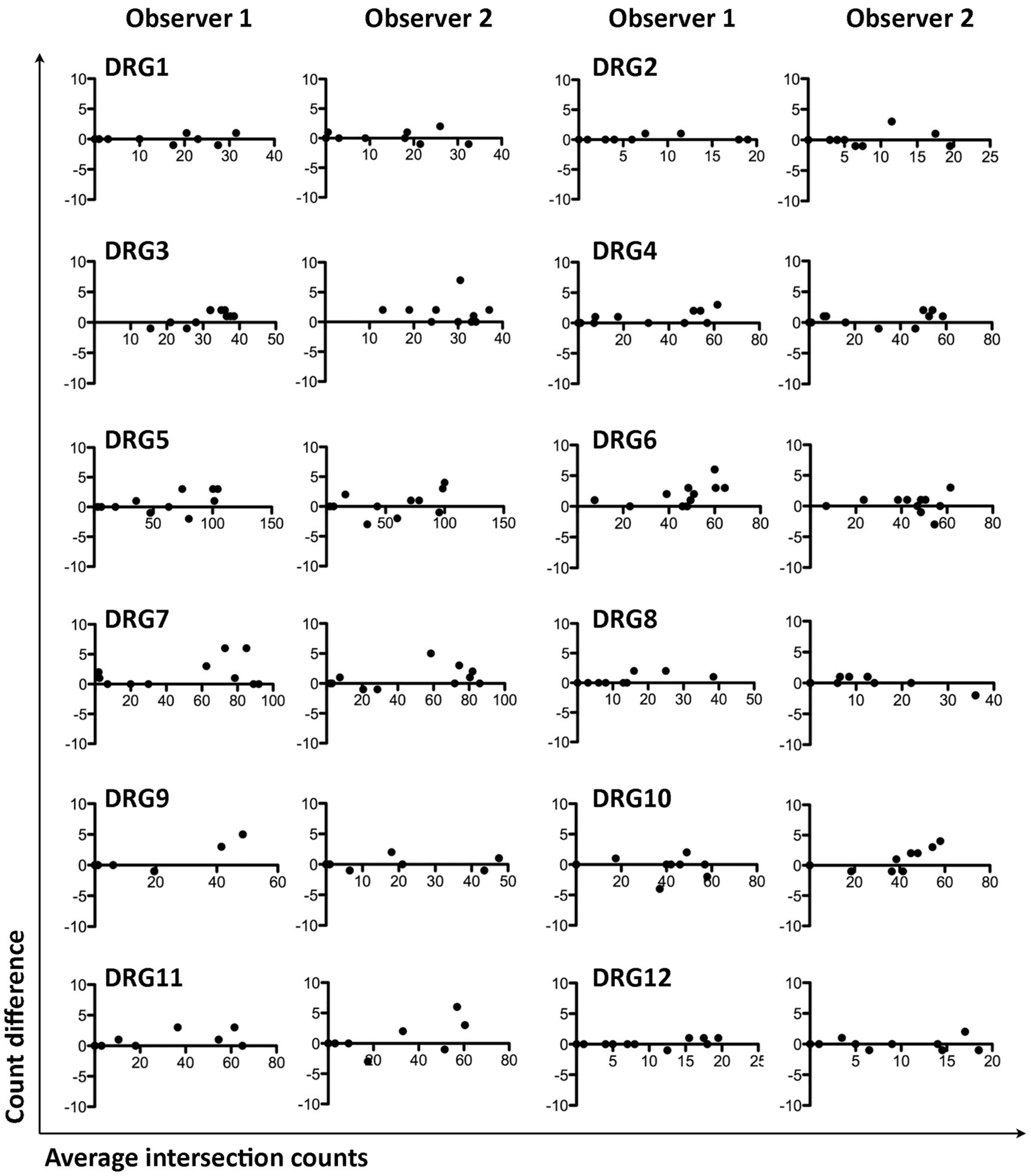

Supplementary Figure 2. Intra-observer agreement for CCIC assay. Comparison by Bland-Altman plots shown for Observer 1 and 2 demonstrated intra-observer agreement with quantification was repeated after 1 week for individual images.

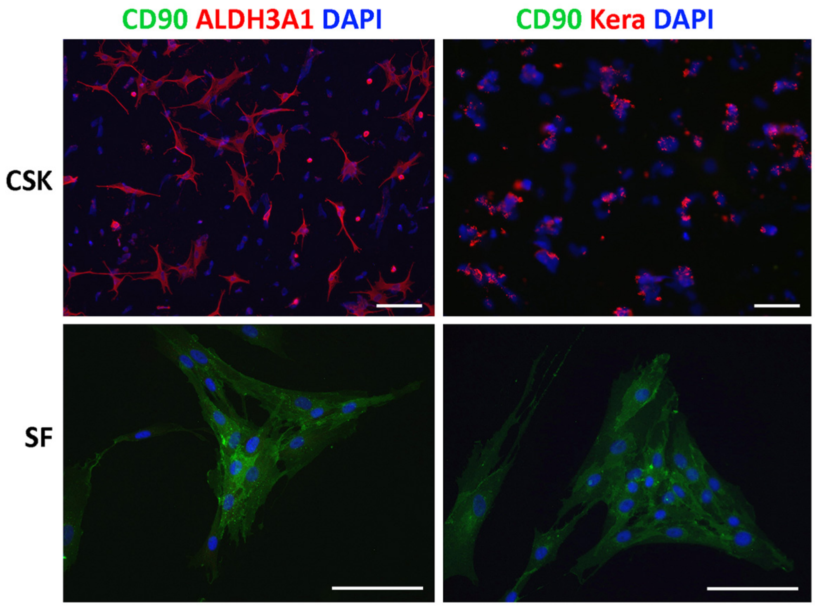

Supplementary Figure 3. Marker expression in CSK and SF cultures after media conditioning. CSK positively expressed ALDH3A1 and Kera (keratocan) but not CD90/Thy1. In contrast, SF expressed CD90 and was negative to CSK markers (ALDH3A1 and Kera). Scale bars: 50 μm.

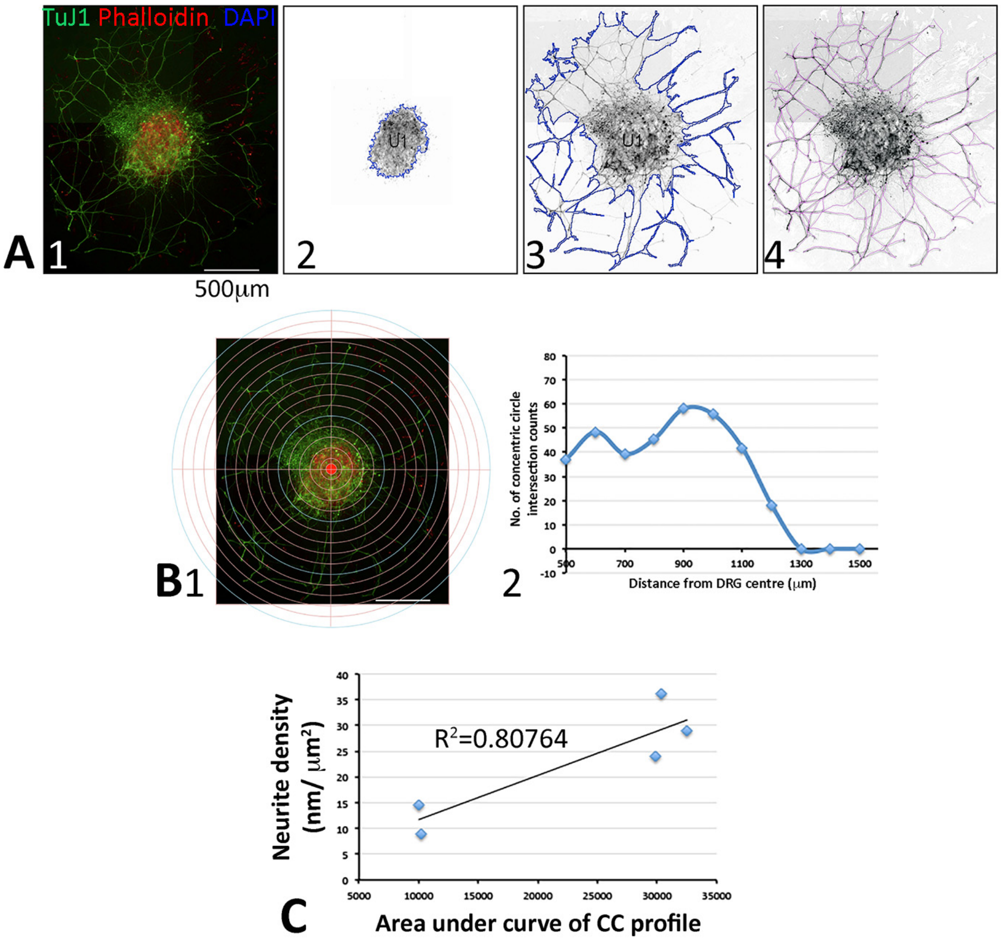

Supplementary Figure 4. Comparison of neurite density assay with concentric circle intersection count method for neurite growth from DRG.

A. Neurite density assay. DRG treated with 50 ng/ml NGF for 72 hours was fixed and processed for co- immunostaining with TuJ1 (green fluorescence) and phalloidin (red fluorescence) (**A1**). Fluorescence image was converted to grey scale and background non-neural cells were eliminated through colour value segmentation. Using Quantity One analysis (BioRad), the DRG tissue (**A2**) and total explant area (**A3**) were contoured and areas were measured. The neurite spanning area was calculated as the total explant area minus DRG tissue area. All extended neurites were traced (**A4**) and the total neurite length was measured by NeuronJ analysis (ImageJ plugin) (National Institute of Health, US). Neurite density was calculated by total

neurite length per unit neurite spanning area and represented as nm length per μm^2 area.

B. Concentric circle intersection count assay. A series of concentric circles with radius increasing at 100 μm and spanning from 500 to 1500 μm range were overlaid on the explant image and aligned to the DRG centre (**B1**). The number of neurites intersecting with each circle line along the distance from DRG centre was manually quantified by two independent observers and each observer counted twice on 2 consecutive days. The neurite extension profile was plotted with the mean number of intersections against distance from the DRG centre (**B2**) and the Area Under Curve (AUC) was calculated.

C. Scatter plot of AUC of CC profile (x-axis) versus neurite density (y-axis) of 5 DRG explants. The best-fit linear line showed $r^2 = 0.80764$, indicating a moderate to high linear correlation of these 2 parameters from independent methods.

Estimates

cat	Mean	Std. Error	95% Wald Confidence Interval Lower	95% Wald Confidence Interval Upper
control	4.03	.479	3.19	5.09
CSK 30%	1.96	.257	1.52	2.54
CSK 60%	2.09	.380	1.46	2.98
CSK 90%	3.56	.664	2.47	5.14
SF 30%	7.50	1.536	5.02	11.20
SF 60%	12.23	2.280	8.49	17.63
SF 90%	15.15	2.295	11.26	20.39

Overall Test Results

Wald Chi-Square	df	Sig.
53.644	6	.000

The Wald chi-square tests the effect of cat. This test is based on the linearly independent pairwise comparisons among the estimated marginal means.

Supplementary Table 1. Independent variance structure with the corrected quasi-likelihood under independence model criterion (QICC) values to compare different CM dosages (the lower QICC the better model fitting) and the estimated marginal means.

Supplementary Table 2. Proteins list identified in conditioned media from human CSKs and SFs by nanoLC-MS/MS analysis

	% Coverage	Accession #	Protein name	Peptides (95%)
A. Proteins commonly found in CSKCM and SFCM				
1	31.4	P04264	KRT1 Keratin 1	29
2	31.7	P13645	KRT10 Keratin 10	27
3	26	P35908	KRT2, Keratin 2	20
4	29.5	P07585	DCN Decorin	15
5	20.1	P36955	SERPINF1 Pigment epithelium-derived factor	9
6	10.8	P08123	COL1A2 Collagen α2(I) chain	12
7	20.4	P51884	LUM Lumican	10
8	24	P01138	NGF Nerve growth factor	12
9	13.7	P02452	COL1A1 Collagen α1(I) chain	18
10	12	P35527	KRT9 Keratin 9	14
11	12.8	O60938	KERA Keratocan	7
12	14.9	P09486	SPARC	6
13	8.9	Q16610	ECM1 Extracellular matrix protein 1	6
14	8	P12109	COL6A1 Collagen α1(VI) chain	8
15	13.3	P01857	IGHG1 Ig γ1 chain C region	4
16	25.8	P41222	PTGDS Prostaglandin-H2 D-isomerase	7
17	19.2	P01034	CST3 Cystatin-C	3
18	3.7	P00751	CFB Complement factor B	3
19	9.6	P01876	IGHA1 Ig α1 chain C region	3
20	7.8	P10909	CLU Clusterin	3
21	3.8	P02768	ALB Serum albumin	2
22	4.3	P35443	THBS4 Thrombospondin-4	3
23	11.6	P05090	APOD Apolipoprotein D	2
24	2.8	P09871	C1S Complement C1s	2
25	9.8	P01859	IGHG2 Ig γ2 chain C region	3
26	0.8	Q99715	COL12A1 Collagen α1(XII) chain	2
27	4	P07477	PRSS1 Trypsin-1	5
28	5.3	P13647	KRT5 Keratin 5	4
29	2.5	P00738	HP Haptoglobin	1
30	2.9	Q9UBP4	DKK3 Dickkopf-related protein 3	1
31	0.6	Q99666	RGPD5 RANBP2-like and GRIP domain-containing protein 5/6	1
32	3.3	Q15113	PCOLCE Procollagen C-endopeptidase enhancer 1	1
33	3.2	Q06828	FMOD Fibromodulin	1

34	10.6	P69905	HBA1 Hemoglobin subunit α	1
35	6.7	P61769	B2M β2-microglobulin	1
36	2.7	P21810	BGN Biglycan	1
37	6.8	P16035	TIMP2 Metalloproteinase inhibitor 2	1
38	4.4	P06733	ENO α-enolase	1
39	2.2	P05156	CFI Complement factor I	1
40	12.7	P24821	TNC Tenascin C	1
41	5.8	P01033	TIMP1 Metalloproteinase inhibitor 1	1
42	3	P00338	LDHA L-lactate dehydrogenase A chain	1
43	0.3	P12111	COL6A3 Collagen α3(VI) chain	1
B. Proteins in CSKCM only				
1	12.3	P02533	KRT14 Keratin 14	6
2	3.5	Q14624	ITIH4 Inter-α-trypsin inhibitor heavy chain H4	2
3	1.8	P08582	MFI2 Melanotransferrin	3
4	1.5	Q9P1V8	SAMD15 Sterile α motif domain-containing protein 15	1
5	0.8	P49917	LIG4 DNA ligase 4	1
6	0.9	P08603	CFH Complement factor H	1
C. Proteins in SFCM only				
1	14.8	P02751	FN1 Fibronectin	32
2	17.6	P05997	COL5A2 Collagen α2(V) chain	35
3	33.5	P08670	VIM Vimentin	14
4	16.8	Q15582	TGFBI TGFβ-induced protein ig-h3	11
5	31.1	P18065	IGFBP2 Insulin-like growth factor-binding protein 2	12
6	17.1	Q02818	NUCB1 Nucleobindin-1	8
7	8.3	P12110	COL6A2 Isoform 2C2A of Collagen α2(VI) chain	9
8	14.6	P08253	MMP2 72 kDa type IV collagenase	7
9	5.8	P07996	THBS1 Thrombospondin-1	6
10	9	P48668	KRT6C Keratin 6C	6
11	15.6	Q12841	FSTL1 Follistatin-related protein 1	6
12	8.9	O43852	CALU Calumenin	4
13	4.6	O94985	CLSTN1 Calsyntenin-1	4
14	21.3	P22692	IGFBP4 Insulin-like growth factor-binding protein 4	6
15	11.8	P08779	KRT16 Keratin 16	6
16	9.9	Q9Y240	CLEC11A C-type lectin domain family 11 member A	3

17	7.9	Q9UBR2	CTSZ Cathepsin Z	4
18	8.3	sQ92743	Serine protease HTRA1	3
19	0.9	P98160	HSPG2 Heparan sulfate proteoglycan core protein	3
20	10.3	P24593	IGFBP5 Insulin-like growth factor-binding protein 5	3
21	2.6	P14543	NID1 Nidogen-1	3
22	9	O43827	ANGPTL7 Angiopoietin-related protein 7	3
23	8.3	O95965	ITGBL1 Integrin β-like protein 1	3
24	12.3	P20366	TAC1, Tachykinin precursor 1	3
25	8.5	P06753-6	TPM3 Isoform 6 of Tropomyosin α3 chain	3
26	5.1	P00736	C1R Complement	5
27	4.4	O00391	QSOX1 Sulfhydryl oxidase 1	3
28	9.4	P26022	PTX3 Pentraxin-related protein	3
29	3.3	Q8TB73	NDNF	2
30	1.9	Q8IUX7	AEBP1 Adipocyte enhancer-binding protein 1	2
31	2.6	Q7Z7G0	ABI3BP Target of Nesh-SH3	2
32	4.7	P05121	SERPINE1 Plasminogen activator inhibitor 1	2
33	35.9	P01834	IGKC Ig kappa chain C region	2
34	7.3	P01308	INS Insulin	3
35	3.3	O60568	PLOD3 Procollagen-lysine,2-oxoglutarate 5-dioxygenase 3	2
36	1.9	Q13822	ENPP2 Ectonucleotide pyrophosphatase/phosphodiesterase family member 2	2
37	9.4	P63104	YWHAZ 14-3-3 protein zeta/delta	2
38	5.3	P02461	COL3A1 Collagen α1(III) chain	8
39	8.5	P67936	TPM4 Tropomyosin α4 chain	3
40	2	P35442	THBS2 Thrombospondin-2	2
41	6.7	P62937	PPIA Peptidyl-prolyl cis-trans isomerase A	1
42	5.5	P55001	MFAP2 Microfibrillar-associated protein 2	1
43	1.1	P11047	LAMC1 Laminin subunit γ1	1
44	2.3	Q9P0M2	AKAP7 A-kinase anchor protein 7 isoform γ	1
45	0.3	Q9NYQ8	FAT2 Protocadherin Fat 2	1
46	3.7	Q9NRN5	OLFML3 Olfactomedin-like protein 3	1
47	3.2	Q9GZP0	PDGFD Platelet-derived growth factor	1

			D	
48	3.3	Q9BRK5	SDF4 45 kDa calcium-binding protein	1
49	3.3	Q96CG8	CTHRC1 Collagen triple helix repeat-containing protein 1	1
50	1.5	Q96AY3	FKBP10 Peptidyl-prolyl cis-trans isomerase	1
51	5.3	Q92520	FAM3C	1
52	0.9	Q8TCG1	KIAA1524 Protein CIP2A	1
53	2.2	Q8NBJ4	GOLM1 Golgi membrane protein 1	1
54	2.4	Q6SA08	TSSK4 Testis-specific serine/threonine-protein kinase 4	1
55	0.6	Q14767	LTBP2 Latent-TGFβ-binding protein 2	1
56	3	Q12805	EFEMP1 EGF-containing fibulin-like extracellular matrix protein 1	1
57	2.1	Q08380	LGALS3BP Galectin-3-binding protein	1
58	5.5	Q06830	PRDX1 Peroxiredoxin-1	1
59	1.5	Q02809	PLOD1 Procollagen-lysine,2-oxoglutarate 5-dioxygenase 1	1
60	2.1	P80303	NUCB2 Nucleobindin-2	1
61	6.6	P61916	NPC2 Epididymal secretory protein E1	1
62	4.2	P60174	TPI1 Triosephosphate isomerase	1
63	2.1	P51888	PRELP Prolargin	1
64	0.2	P35556	FBN2 Fibrillin-2	1
65	5.7	P31946	YWHAB 14-3-3 protein beta/alpha	1
66	1.3	P30530	AXL Tyrosine-protein kinase receptor UFO	1
67	2.8	P30101	PDIA3 Protein disulfide-isomerase A3	1
68	1.4	P29401	TKT Transketolase	1
69	2.9	P29279	CTGF Connective tissue growth factor	1
70	5.4	P24592	IGFBP6 Insulin-like growth factor-binding protein 6	1
71	4.2	P23284	PPIB Peptidyl-prolyl cis-trans isomerase B	1
72	2	P23142	FBLN1 Fibulin-1	1
73	3	P20774	OGN Mimecan	1
74	1.3	P16070	CD44	1
75	2.2	P11279	LAMP1 Lysosome-associated membrane glycoprotein 1	1
76	14.2	P0CG06	IGLC3 Ig lambda-3 chain C regions	1
77	11.1	P09382	LGALS1 Galectin-1	1

78	10.7	P08493	MGP Matrix Gla protein	1
79	4.5	P04406	GAPDH Glyceraldehyde-3-phosphate dehydrogenase	1
80	6.5	P00441	SOD1 Superoxide dismutase [Cu-Zn]	1
81	1.8	O95967	EFEMP2 EGF-containing fibulin-like extracellular matrix protein 2	1
82	3	O14498	ISLR Immunoglobulin superfamily containing leucine-rich repeat protein	1
83	2.4	O00622	CYR61	1
84	0.6	Q9ULK4	MED23 Mediator of RNA polymerase II transcription subunit 23	1
85	1.2	Q14246	ADGRE1 Adhesion G protein-coupled receptor E1	1
86	12.3	P63261	ACTG1 Actin γ1	3
87	10.3	P23083	Ig heavy chain V-I region V35	1
88	1.3	Q5T3F8	TMEM63B CSC1-like protein 2	1

Supplementary Table 3. Donor cornea information

**Donor**	**Age**	**Gender**	**Cause of death**	**Preservation in Optisol before cell culture (days)**
1	13	F	Gun shot death	12
2	21	M	Bowel obstruction	9
3	18	M	Pulmonary fibrosis	9
4	64	F	Heart failure	8
5	50	M	Hepatocellular cancer	6
6	35	M	Aspiration pneumonia	8

F: female; M: male

Supplementary Table 4. Primary antibodies used in this study

Antibody	Source	Application	Remarks
TuJ1	Santa Cruz	0.5 μg/ml	
Lumican	Sigma	1 μg/ml	Endo-β-galactosidase treatment
ALDH3A1	Proteintech	1 μg/ml	Methanol pre-treatment
CD90 (Thy1)	Millipore	1 μg/ml	
Fibroblast 5B5	Novus Biol	0.5 μg/ml	
α-SMA	DAKO	1 μg/ml	Ice-cold acetone fixation
Fluorescein conjugated-phalloidin	Invitrogen	0.5 μg/ml	

Supplementary Table 5. Gene expression primers

	Gene	GeneBank Accession No.	Sequences (5'-3')	Product size (bp)
1	AcvR1	NM_001105.4	F: CTGGCCAAGCCGTGGAGTGCTGCC<br>R: GTACTGGAGTGTCTTGATGTCATG	571
2	BGN	NM_001711.4	F: GAGGACCTGCTTCGCTACTC<br>R: CCCTGGCCAACTTGTTGTTG	136
3	ENO2	NM_001975.2	F: GTGTCTCTGGCCGTGTGTAA<br>R: TCTCCAGGATATTGGGGGCA	319
4	GAPDH	NM_002046.3	F: GAACATCATCCCTGCATCCA<br>R: CCAGTGAGCTTCCCGTTCA	226
5	IL4	BC_067514.1	F: ACTTTGAACAGCCTCACAGAG<br>R: TTGGAGGCAGCAAAGATGTC	172
6	ITGA5	NM_002205.3	F: TGCTGGACTGTGGAGAAGACAACA<br>R: TCTGGGCATGGAAAGTGAGGTTCA	120
7	KERA	NM_007035.3	F: CAGGAGAAACAGGGCCTACAGA<br>R: TCCAGCTTGGGGTCCTGAA	191
8	LUM	NM_002345.3	F: CCTGGTTGAGCTGGATCTGT<br>R: TGGTTTCTGAGATGCGATTG	194
9	MCP1/CCL2	NM_002982.3	F: GCCTCCAGCATGAAAGTCTC<br>R: AGGTGACTGGGGCATTGAT	99
10	MMP2	NM_004530.5	F: GGCCCTGTCACTCCTGAGAT<br>R: GGCATCCAGGTTATCGGGGA	437
11	Sema3f	NM_004186.3	F: AGCAGACCCAGGACGTGAG<br>R: AAGACCATGCGAATATCAGCC	145
12	TAC/SP	NM_013996.2	F: GGTACGACAGCGACCAGATCA<br>R: CCCGTTTGCCCATTAATCCA	112
13	TGFBI	NM_000660.5	F: CCCAGCATCTGCAAAGCTC<br>R: GTCAATGTACAGCTGCCGCA	101
14	TIMP1	NM_003254.2	F: TTTTGTGGCTCCCTGGAACA<br>R: GGACTGGAAGCCCTTTTCAGA	184
15	TNC	NM_002160.3	F: GGCTACCGATGGGATCTTCG<br>R: GTCCAGCAGCTTCCCAGAAT	337
